# Fetal autonomic brain age scores, segmented heart rate variability analysis, and traditional short term variability

**DOI:** 10.3389/fnhum.2014.00948

**Published:** 2014-11-25

**Authors:** Dirk Hoyer, Eva-Maria Kowalski, Alexander Schmidt, Florian Tetschke, Samuel Nowack, Anja Rudolph, Ulrike Wallwitz, Isabelle Kynass, Franziska Bode, Janine Tegtmeyer, Kathrin Kumm, Liviu Moraru, Theresa Götz, Jens Haueisen, Otto W. Witte, Ekkehard Schleußner, Uwe Schneider

**Affiliations:** ^1^Biomagnetic Center, Hans Berger Department of Neurology, Jena University Hospital, Friedrich Schiller UniversityJena, Germany; ^2^Institute of Biomedical Engineering and Informatics, Ilmenau University of TechnologyIlmenau, Germany; ^3^Department of Obstetrics, Jena University Hospital, Friedrich Schiller UniversityJena, Germany

**Keywords:** prenatal diagnosis, fetal autonomic brain age, magnetocardiography, cardiotocography

## Abstract

Disturbances of fetal autonomic brain development can be evaluated from fetal heart rate patterns (HRP) reflecting the activity of the autonomic nervous system. Although HRP analysis from cardiotocographic (CTG) recordings is established for fetal surveillance, temporal resolution is low. Fetal magnetocardiography (MCG), however, provides stable continuous recordings at a higher temporal resolution combined with a more precise heart rate variability (HRV) analysis. A direct comparison of CTG and MCG based HRV analysis is pending. The aims of the present study are: (i) to compare the fetal maturation age predicting value of the MCG based fetal Autonomic Brain Age Score (fABAS) approach with that of CTG based Dawes-Redman methodology; and (ii) to elaborate fABAS methodology by segmentation according to fetal behavioral states and HRP. We investigated MCG recordings from 418 normal fetuses, aged between 21 and 40 weeks of gestation. In linear regression models we obtained an age predicting value of CTG compatible short term variability (STV) of *R*^2^ = 0.200 (coefficient of determination) in contrast to MCG/fABAS related multivariate models with *R*^2^ = 0.648 in 30 min recordings, *R*^2^ = 0.610 in active sleep segments of 10 min, and *R*^2^ = 0.626 in quiet sleep segments of 10 min. Additionally segmented analysis under particular exclusion of accelerations (AC) and decelerations (DC) in quiet sleep resulted in a novel multivariate model with *R*^2^ = 0.706. According to our results, fMCG based fABAS may provide a promising tool for the estimation of fetal autonomic brain age. Beside other traditional and novel HRV indices as possible indicators of developmental disturbances, the establishment of a fABAS score normogram may represent a specific reference. The present results are intended to contribute to further exploration and validation using independent data sets and multicenter research structures.

## Introduction

Prenatal risk factors can permanently change the fetal brain development and are associated with diseases in later age. Adverse influences during fetal development that became permanently programmed can increase the postnatal risk for cardiovascular, metabolic, hyperkinetic, cognitive, learning and behavioral disorders (e.g., Barker, 1998, 2002; Barker et al., 2002; O’Keeffe et al., 2003; Van den Bergh et al., 2005).

Prenatal functional diagnosis is limited and requires innovative concepts. In that context, the fetal autonomic (neuro-vegetative) control plays a key role since it provides valuable information about several control systems that are mediated by the autonomic nervous system. The associated heart rate patterns (HRP) are one of the few signals that can be obtained non-invasively from the fetus, and hence, heart rate variability (HRV) analysis is uniquely suited to assess the fetal functional autonomic brain development. Fetal HRP provide quantitative information about sympathetic and vagal activation, fetal behavioral states, and fetal movements (Nijhuis et al., 1982; Van Leeuwen et al., 1999; David et al., 2007; Hoyer et al., 2009; Schmidt et al., 2014).

In order to obtain normal values of maturation indices in healthy fetuses and deviations due to risk factors, a sophisticated analysis of fetal HRP is required. Finally, the association between prenatal and postnatal autonomic control and disturbances needs to be investigated in that context.

Antepartum cardiotocography (CTG) is a predominant established method that contributes to fetal surveillance and risk assessment both antenatal and during labor based on the analysis of fetal HRP age (Nijhuis et al., 2000; Pardey et al., 2002; Serra et al., 2008, 2009). FIGO (International Federation of Gynaecology and Obstetrics) risk score as well as the fluctuation indices short term variability (STV) and long term variability (LTV) are realized in Dawes-Redman methodology (Pardey et al., 2002). The fetal HRP displayed in CTG depends on gestational age, fetal activity and a variety of other factors, but classification of recordings primarily aimed on distinguishing the healthy from the distressed fetus rather than precisely assessing maturation. Hence, the resulting question to be followed in this study is, to which extent CTG methodology allows a fetal autonomic brain age assessment in comparison to the higher quality MCG methodology.

Universal characteristics of evolution and development in non-living and living nature are increasing fluctuation amplitude, increasing complexity and pattern formation. Those characteristics similarly apply in the phylogenetic and the ontogenetic development. The fetal HRP reflect corresponding characteristics of the maturating fetal autonomic brain activity. All of those characteristics can be interpreted as reflecting autonomous influences of the sympathetic and parasympathetic nervous systems, influenced by the superordinate medullary centers. In a previous study, using continuous magnetocardiographic (MCG) recordings at 1 ms temporal resolution, we designed a resulting “fetal autonomic brain age score” (fABAS; Hoyer et al., 2013b).

However, a certain part of variance could not be explained by the score and the question arises how and to which extent a more sophisticated model may improve the performance. In that context, segmentation of the measured HRP under consideration of behavioral states as well as of the particular exclusion of acceleration (AC) and deceleration (DC) patterns may provide information about different aspects of autonomic modulations in more detail.

The aims of the present study are: (i) to compare the fetal maturation age predicting value of the MCG based fABAS approach with that of Dawes-Redman methodology; and (ii) to elaborate fABAS methodology by segmentation of the recordings according to behavioral states and HRP.

## Materials and methods

### Subjects and data aquisition

From the study database of the Biomagnetic Center, Department of Neurology, and Department of Obstetrics, both Jena University Hospital, recordings of 418 normal singleton fetuses, aged between 21 and 40 weeks of gestation (WGA), healthy according to standard obstetric observation methods, single recording in a non-stress situation were included. The study was approved by the Local Ethics Committee of the Friedrich Schiller University. All women signed a written, informed consent form.

All measurements were taken in a magnetically shielded room at the Biomagnetic Center, Department of Neurology, Jena University Hospital using the vector-magnetograph ARGOS 200 (ATB, Chieti, Italy). Pregnant women were positioned supine or with a slight twist to either side to prevent compression of the inferior vena cava. The dewar was positioned as close as possible above the fetal heart determined by sonographic localization, but without contact to the maternal abdominal wall. The MCG signal was recorded over a period of 30 min with a sampling rate of 1024 Hz. The fetal heart beats were detected using a newly developed independent component analysis based strategy (Schmidt et al., 2014). The fetal body movements were reconstructed from the fMCG signal (for details see Schmidt et al., 2014).

### CTG compatible analysis according to Dawes-Redman

In the present work the heart beat intervals (MCG sampling period ≈1 ms) were integrated over 3.75 s epochs in order to obtain a CTG compatible signal of the identical recording. Analyzable 1 min sections were considered as those that contain neither large decelerations nor more than 50% artifacts (not detected beats/beat intervals; Pardey et al., 2002).

- STV is calculated as mean difference between consecutive heart beat interval epochs in all analyzable 1 min sections. The results of all analyzed 1 min sections are averaged.
- LTV is calculated as fluctuation range of heart beat interval epochs in analyzable 1 min sections. The fluctuation range is calculated as a sum between maximal deviation above baseline and maximal deviation below baseline. The fluctuation ranges of all analyzed 1 min sections are averaged.

Furthermore, the Dawes-Redman criteria for normality from 26 WGA upwards, that require up to 60 min recordings, were formally applied to the 30 min recordings of the fetuses aged at least 26 WGA (*n* = 313). The following criteria have to be met (adapted from Pardey et al., 2002):

- The recording must contain at least one episode of high variation.
- STV >3.0 ms, but if it is <4.5 ms LTV averaged across all episodes of high variation must be >3rd percentile for WGA.
- No evidence of a high-frequency sinusoidal rhythm.
- At least one AC, or a fetal movement rate of ≥20 per hour and a LTV averaged across all episodes of high variation >10th percentile for WGA.
- At least one fetal movement or three AC.
- No DC of >20 lost beats if the recording is <30 min, no more than one DC of 21–100 lost beats if it is >30 min, and no DC at all >100 lost beats.
- The basal fetal heart rate must be 116–160 beats/min if the recording is <30 min.
- LTV within 3 SD of its estimated value or (a) STV >5.0 ms; (b) an episode of high variation with ≥0.5 fetal movements per minute; (c) the basal fHR ≥120 beats/min; and (d) the signal loss <30%.
- The final epoch of the recording must not be part of a DC.
- No suspected artifacts at the end of the recording if the recording is <60 min.

In that context, an AC is defined as an increase in heart rate for more than 15 s with a minimum deviation from baseline exceeding 10 bpm. A DC is defined as a decrease below the baseline for more than 30 s and a deviation >20 bpm or 60 s and >10 bpm, respectively (Pardey et al., 2002).

### MCG based heart rate variability analysis—fetal autonomic brain age score

The fABAS was previously proposed by the authors using a calculation precision of ≈1 ms according to the MCG sampling rate (Hoyer et al., 2013b). Fetal autonomic brain age score based on particular HRV parameters that were selected according to universal developmental characteristics, namely increasing fluctuation amplitude (assessed by AMP), increasing complexity (assessed by gMSE3), and pattern formation (assessed by skewness, pNN5, lnVLF/LF, see Table 1). In that previous work, the fetal age was predicted by multivariate linear regression models (forward procedure: stepwise inclusion of variables while *P*(*F*) < 0.05; backward procedure: stepwise exclusion of variables while *P*(*F*) > 0.1) for each sleep state independently. The resulting models for quiet and active sleep were considered fABAS. While [gMSE3, skewness, VLF/LF, pNN5] contributed in the quiet sleep model, [AMP, skewness, gMSE3, pNN5, VLF/LF] contributed in the active sleep model. Here, an additional model was built for the entire 30 min recordings that can include only one or several behavioral states. The resulting additional model constitutes a third branch of fABAS. Furthermore, the linear regression models were supplemented with quadratic term regression models that were built using the same HRV parameters.

**Table 1:** **Heart rate variability indices**.

Parameter	Meaning, Interpretation	Calculation
**Dawes-Redman**
STV	**Short Term Variability** Integrative sympatho-vagal modulations, fluctuation periods of seconds	Mean difference between consecutive heart beat interval epochs of 3.75 s, w/o DC and artifacts	#x0003C;50%
LTV	**Long Term Variability** Fluctuation periods of minutes	Mean fluctuation range of heart beat interval epochs in 1 min sections, w/o DC and artifacts <50%
**fABAS**
AMP	**Fluctuation range** of heart beat intervals, overall sympatho-vagal modulations	20–95 inter-quantile distance of detrended NN interval series
gMSE3	**Complexity** of sympatho-vagal rhythms	Generalized Mutual Information at coarse graining level 3 of beat interval series, see Hoyer et al. (2013a)
skewness	**Asymmetry**, contribution of vagal and sympathetic activity with their different time constants, decline of DC and formation of AC	skewness of instantaneous heart rate series
pNN5	**Fast, vagal** Mainly vagally modulated rhythms	Percentage of differences between adjacent NN intervals that are >5 ms.
lnVLF/LF	**Baseline fluctuation** in relation to sympatho-vagal modulations	Ratio between very low (0.02–0.08 Hz) and low (0.08–0.2 Hz) frequency band power
**Pattern segmented**
gMSE3 w/o DC
gMSE3 basic	**w/o DC** - parameters under exclusion of DC	Mean of parameter of subsegments without DC
skewness w/o DC
skewness basic
pNN5 w/o DC	**basic** - parameters of basic rhythm	Mean of parameter of subsegments w/o DC and w/o AC
pNN5 basic
lnVLF/LF w/o DC
lnVLF/LF basic

* Segmented analysis of fluctuation range (AMP) was not performed due to lacking significance in quiet sleep. Partly adapted from Hoyer et al. (2013b), further details in TaskForce (1996), Pardey et al. (2002), Hoyer et al. (2013a).

### Sleep state segmentation

From the entire 30 min recordings 10 min segments according to quiet and active sleep related HRP I, HRP II were selected following a consensus decision by three independent obstetricians according to an advanced version of standard criteria (Nijhuis et al., 1982; Schneider et al., 2009; Hoyer et al., 2013b).

- HRP I (quiet state, correlated to quiet sleep 1F): stable fetal heart rate (fHR) (variation of visually determined floating baseline <10 bpm/3 min) with a small oscillation bandwidth (< ±5 bpm from floating baseline fHR), isolated (maximum 2 per 10 min) AC (>15 bpm over >15 s) and a floating baseline fHR that does not exceed 160 bpm.
- HRP II (active state, correlated to active sleep 2F): fluctuating fHR with an oscillation bandwidth exceeding +/− 5 bpm from floating baseline, frequent (at least 3 per 10 min) AC (>15 bpm, >15 s), and the fHR exceeding 160 bpm only during AC.
- HRP III (active state, correlated to active awakeness 4F): fHR patterns with long-lasting AC exceeding 160 bpm, frequently fused into a sustained tachycardia.

Based on the classification results we selected 10 min sections of quiet sleep (*n* = 137) and active sleep (*n* = 344). Consequently, in 63 of the 418 recordings, sections of both, HRP I and HRP II, were classified. Active awakeness classified recordings were not analyzed because of their small sample size (*n* = 29).

### Pattern segmentation–partial exclusion of heart rate accelerations and decelerations

In addition, periods without DC and segments of basic activity (with neither DC nor AC), respectively, were analyzed in the HRP I segments. The methodology of the here proposed MCG based segmented HRV analysis is different from the CTG—Dawes-Redman methodology used above. In the present context, the definitions of AC and DC were modified according to the following rules:

- Sections without DC were identified in moving 3 min windows (shifted by 1 min over 10 min segment) if there were no deviations below the baseline (estimated for each 3 min window) > 10 bpm;
- Sections of basic activity were identified in moving 3 min windows (shifted by 1 min over 10 min segment) if there were no deviations below or above the baseline (estimated for each 3 min window) > 10 bpm.

The baseline was estimated according to original Dawes-Redman methodology (Pardey et al., 2002). From all identified windows the HRV parameters were calculated and averaged.

This kind of pattern segmentation was performed for all significantly age predicting HRV parameters of fABAS (see Table 1).

**Figure 1:**
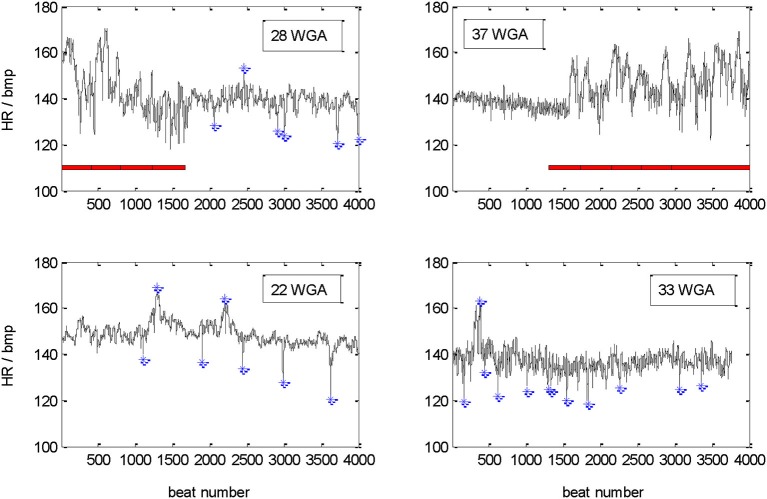
**Tachograms of 30 min recordings of four different fetuses**. Upper part: changes between sections of active (marked by red horizontal bar) and quiet sleep related heart rate patterns in a younger (28 WGA) and an older (37 WGA) fetus. Lower part: quiet sleep related heart rate patterns at 22 and 33 WGA. DC and AC (>10 bpm deviation from floating baseline, marked by blue *) are identified in the quiet sleep sections only.

The example recordings shown in Figure 1 demonstrate the diversity of the recorded pattern. In the upper part a change between active and quiet state in a premature age of 28 WGA is compared with a clear change from quiet to active state in the mature age of 37 WGA. In the part underneath, recordings classified as quiet sleep at 22 and 33 WGA are shown. Please notice partly unstable baseline, AC and DC are marked in the quiet sleep section. There are neither periods of incorrect heart beat detections (beat intervals) nor periods of dropouts.

### Statistical analysis

The predictive value of the HRV parameters was assessed by univariate and multivariate, linear and quadratic term regression models over the entire investigated maturation period (corrected coefficient of determination *R*^2^). The cases were weighted to approximate equal distribution over gestational age. *P* < 0.05 was considered significant. Since most of the predictors were significant, only non-significant results are marked by “n.s.”. For better reading, predictors with *R*^2^ > 0.3 were marked by bold letters. All statistical analyses were carried out using IBM SPSS Statistics 21.

## Results

### 30 min recordings

**Figure 2:**
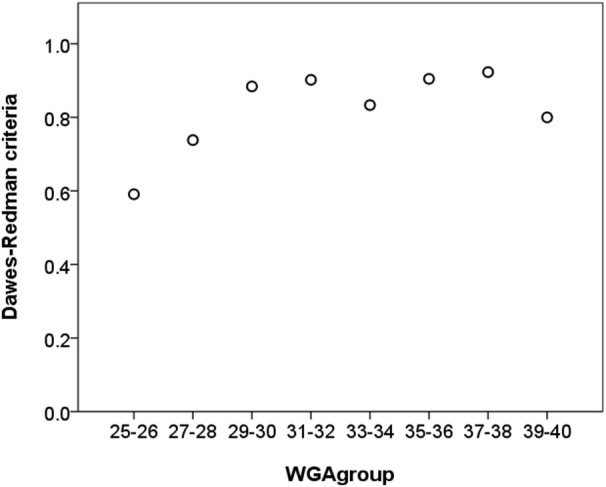
**Relative frequency of recordings that meet the Dawes-Redman criteria (1 = 100%) vs. chronological age (in weeks GA) of 30 min recordings**. In absolute values, 134 of 167 cases met the criteria in the subset of ≤32 WGA and 130 of 146 in the subset of >32 WGA, respectively.

Traditional STV and LTV were calculated from analyzable periods according to CTG methodology like outlined above. Short term variability predicted the maturation age with a low coefficients of determination (*R*^2^ = 0.200, 206, linear and quadratic model, Table 2). The Dawes-Redman criteria were met with increasing frequency between 26 and 32 WGA and were consistently met from 32 WGA onwards (Figure 2).

**Table 2:** **Analyses of 30 min recordings: univariate and multivariate, linear and quadratic term regression models, coefficients of determination *R*^2^, parameters selected according to CTG (Dawes-Redman related (Pardey et al., 2002)) and MCG based fABAS related (Hoyer et al., 2013b), *R*^2^ > 0.3 in bold, all cases significant**.

Parameter	30 min recording	
	Linear	Quadratic
**CTG compatible**
STV (ms)	0.200	0.206
LTV (ms)	0.085	0.116
**fMCG based, fABAS related**
**Time domain**
AMP	**0.312**	**0.321**
skewness	**0.458**	**0.502**
pNN5	**0.347**	**0.367**
**Power spectra**
lnVLF/LF	0.034	0.060
**Complexity**
gMSE3	0.226	0.231
**Multivariate model, fABAS**
[AMP, skewness, pNN5, lnVLF/LF, gMSE3]	**0.648**	**0.656**
**Multivariate model, fABAS + STV**
[AMP, skewness, pNN5, lnVLF/LF, gMSE3, STV]	**0.649**	**0.657**

**Figure 3:**
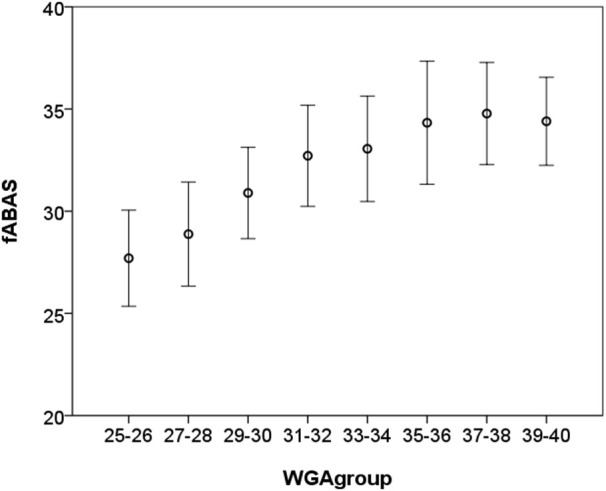
**Fetal autonomic brain age score [AMP, skewness, pNN5, lnVLF/LF, gMSE3] vs. chronological age (in WGA) of 30 min recordings, mean ± standard deviation**.

From the fABAS related HRV parameters, AMP, skewness, and pNN5 and provided strong univariate linear age predictors (*R*^2^ = 0.312, 0.458, 0.347). They were partly improved by including a quadratic term (*R*^2^ = 0.321, 0.502, 0.367). In the multivariate models all parameters significantly contributed leading to *R*^2^ = 0.648 and *R*^2^ = 0.656, respectively (Table 2, Figure 3). In contrast, STV and LTV did not provide additional predictive value to the multivariate models.

### Segmented recordings

#### Active sleep 10 min segments

In the active sleep data, AMP (*R*^2^ = 0.392 and 0.430, linear and quadratic), skewness (*R*^2^ = 0.352 and 0.431) and pNN5 (*R*^2^ = 0.350 and 0.376) clearly predicted the fetal maturation age. In the multivariate models, additionally gMSE3 and lnVLF/LF contributed leading to *R*^2^ = 0.610 and 0.636 in the linear and quadratic, respectively, models (Table 3).

**Table 3:** **Analyses of 10 min segments in active sleep: linear and quadratic regression models, coefficients of determination *R*^2^, parameters selected according to fABAS (Hoyer et al., 2013b), *R*^2^ > 0.3 in bold**.

Predictor	*R*^2^	
	Linear	Quadratic
**Time domain**
AMP	**0.392**	**0.430**
Skewness	**0.352**	**0.431**
pNN5	**0.350**	**0.376**
**Power spectra**
lnVLF/LF	0.098	0.103
**Complexity**
gMSE3	0.128	0.130
**Multivariate model**	-	-
[AMP, Skewness, gMSE3, pNN5, lnVLF/LF]	**0.610**	**0.636**

#### Quiet sleep 10 min segments

In the quiet sleep data, gMSE3 (*R*^2^ = 0.542 and 0.565), but also pNN5 (*R*^2^ = 0.320 and 0.338) and skewness (*R*^2^ = 0.316 and 0.320) were stronger predictors than lnVLF/LF (*R*^2^ = 0.147 and 0.159). AMP did not provide predictive value. The stepwise built multivariate model included [skewness, gMSE3, pNN5] (*R*^2^ = 0.626 and 0.641; Table 4).

**Table 4:** **Analyses of 10 min segments in quiet sleep: additional pattern segmentation (without decelerations: w/o DC; basic activity, neither DC nor AC: basic), linear and quadratic regression models, coefficients of determination *R*^2^, parameters according to Table 2, *R*^2^ > 0.3 in bold**.

Predictor	*R*^2^	
	Linear	Quadratic
**Time domain**
AMP	n.s.	n.s.
Skewness	**0.316**	**0.320**
skewness w/o DC	0.123	0.127
skewness basic	0.032	0.038
pNN5	**0.320**	**0.338**
pNN5 w/o DC	**0.396**	**0.451**
pNN5 basic	**0.374**	**0.428**
**Power spectra**
lnVLF/LF	0.147	0.159
lnVLF/LF w/o DC	0.192	0.201
lnVLF/LF basic	0.195	0.206
**Complexity**
gMSE3	**0.542**	**0.565**
gMSE3 w/o DC	**0.609**	**0.627**
gMSE3 basic	**0.632**	**0.657**
**Multivariate model, fABAS indices**
[Skewness, gMSE3, pNN5]	**0.626**	**0.641**
**Multivariate model, pattern segmented**
[Skewness, gMSE3, gMSE3w/oDC, gMSE3basic]	**0.706**	**0.714**

#### Further pattern segmentation in quiet sleep

Further pattern segmentation, namely the exclusion of DC and AC removed the predictive value of skewness. This result is consistent with the fact that skewness mainly reflects asymmetries due to DC and AC which are important maturation characteristics. Other aspects of behavior are basic activity, i.e., the basic rhythm containing neither AC nor DC, and activity under exclusion of DC. Concerning those aspects, the predictive value of pNN5, gMSE3 and lnVLF/LF was increased. The gMSE3 related indices were the strongest univariate predictors (gMSE, gMSEw/oDC, gMSE3basic: *R*^2^ = 0.542, 0.609. 0.632 in the linear models, and *R*^2^ = 0.565, 0.627. 0.657 in the quadratic models, see Table 4).

The stepwise built multivariate model resulted on the inclusion of [skewness, gMSE3, gMSEw/oDC, gMSE3basic] with *R*^2^ = 0.706 in the linear and *R*^2^ = 0.714 quadratic model. This result indicates a relevant advantage compared to the consideration of data that are classified as quiet sleep as a total without further pattern segmentation.

## Discussion

Traditional HRV indices reflect: (i) vagal activity during quiet sleep; (ii) sympathetic activity during active sleep; and (iii) integrative control in longer recordings with changing states. In a recent study, we have demonstrated a strong relationship between those HRV indices and fetal maturation age (Hoyer et al., 2009). Compared to those traditional HRV indices, we newly developed a fABAS based on universal developmental characteristics assessing maturation age with a high precision in active and quiet sleep (Hoyer et al., 2013b). In this study we show that fABAS based methodology significantly improves fetal maturation age assessment compared to established CTG. Those results are based on our comprehensive database obtained from MCG measurements.

The particular aims of the present study were: (i) to compare the fetal maturation age predicting value of the MCG based fABAS approach with that of CTG (Dawes-Redman) compatible STV; and (ii) to elaborate fABAS methodology by segmentation according to behavioral states (overall recording, active and quiet sleep segments) and HRP.

### 30 min recordings

The maturation age predicting value of STV (*R*^2^ = 0.200, linear model) was significantly lower in comparison to fABAS.

Interestingly, using the indices of fABAS applied to the entire 30 min recordings, the maturation age was clearly predictable (*R*^2^ = 0.648, linear model), although the signals were very heterogeneous due to the occurrence of only one or several behavioral states. The increased *R*^2^ in the quadratic models, moreover, may reflect the saturating maturation after 32 WGA mainly expressed in skewness (see Figure 3). This characteristic curve is in line with previous results of power spectral analysis (Van Leeuwen et al., 2003).

### Active and quiet sleep 10 min segments

The predictive value of the fABAS related models in active and quiet sleep (*R*^2^ = 0.610, 0.626, linear models) was slightly lower in comparison to the 30 min recordings. However, it should be noticed that state dependent aspects of autonomic control, such as vagal and sympathetic dominance, provided an almost similar predictive value. Saturating maturation after 32 WGA considered in quadratic models of vagal HRV (pNN5) in quiet sleep and mainly sympathetic/overall HRV (AMP, skewness) in active sleep may have also contributed to increased *R*^2^ in the quadratic models.

### Further pattern segmentation in quiet sleep

Further segmentation in quiet sleep focused on the differential contribution of basic activity and AC as well as DC of at least 10 bpm deviation from baseline. The resulting independent factors of the novel model, namely skewness, gMSE3, gMSE3w/oDC, gMSE3basic, may highlight the role of complex adjustments between sympatho-vagally mediated fluctuations in connection with AC and DC patterns. The predictive value (*R*^2^ = 0.706, linear model) was clearly higher than that of fABAS. This result indicates that the essential universal developmental characteristics, namely increasing complexity and pattern formation which go hand in hand with fetal development, also apply for the particular pattern during quiet sleep. Furthermore, the result implies that a consideration of the respective physiological pattern in detail, might even improve the maturation assessment. Therefore, fABAS may serve as classification tool for the general condition, whereas further pattern segmentation provides a more detailed focus on an observed abnormality.

**Figure 4:**
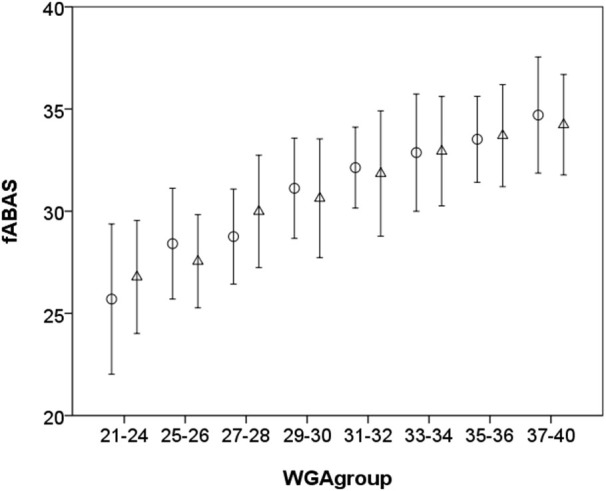
**Fetal autonomic brain age score [Skewness, gMSE3, pNN5] for quiet sleep segments ○ and [AMP, Skewness, gMSE3, pNN5, lnVLF/LF] for active sleep segments Δ vs. chronological age (in WGA), mean ± standard deviation, notice that 21–24 WGA and 37–40 WGA, respectively, are merged due to the small number of samples**.

Fetal autonomic brain age score indices in combination with the novel pattern segmentation based on our comprehensive MCG database can serve as a representative norm-sample of the normal fetal autonomic maturation age (see Figures 3, 4). With that it will be easy to detect even minor deviations from the normal fetal development.

### Methodological issues

In principle, the application of the Dawes-Redman criteria and LTV as a traditional CTG methodology to MCG based HRV analysis was possible. During a period of 60 min, which is recommended for the application of the Dawes-Redman criteria, at least one active pattern is statistically expected to occur. However, since only 30 min recordings were analyzed, a number of subjects did not meet the criteria. Recordings of 60 min length would allow direct consideration of FIGO Dawes-Redman criteria. Even longer observations would furthermore lead to a better evaluation of sleep state dynamics. However, due to patient compliance and the possibly increasing number of artifacts in longer measurements, the performance in shorter recordings is of particular interest.

The segmentation according to behavioral state classification is the result of a consensus decision of three experts based on HRP that furthermore slightly change with maturation age between 21–40 WGA. This classification is not always unequivocal, but it reflects the heterogeneity of recordings and the state of the art.

The present results are mainly representative for the investigated 30 min recordings and 10 min segments. A next necessary step would be the validation and improvement of the presented methodology in recordings from different measurement sites.

For a fair evaluation of the here proposed methodology and search for the optimum, other HRV indices should also be taken into consideration. The here presented methodology allows the inclusion of further precise MCG HRV indices as well of FIGO recommended CTG compatible indices.

In the subsequent steps, multifactorial models are required to consider both, maturation age and developmental disorders.

## Conclusion

Heart rate variability indices selected according to fABAS using 30 min fMCG recordings and segmented HRV analysis provide a promising tool for the estimation of the fetal autonomic brain age that is superior to CTG based indices. Resulting normograms of normal autonomic brain maturation may constitute significant references for the identification of developmental disturbances. The presented methodology is intended to contribute to further exploration and validation with regard to the early identification of developmental disorders using independent data sets in multicenter studies.

## Conflict of interest statement

The authors declare that the research was conducted in the absence of any commercial or financial relationships that could be construed as a potential conflict of interest.
